# Honey bees preferentially consume freshly-stored pollen

**DOI:** 10.1371/journal.pone.0175933

**Published:** 2017-04-21

**Authors:** Mark J. Carroll, Nicholas Brown, Craig Goodall, Alexandra M. Downs, Timothy H. Sheenan, Kirk E. Anderson

**Affiliations:** 1 Carl Hayden Bee Research Center USDA-ARS, Tucson, Arizona, United States of America; 2 Department of Entomology, University of Arizona, Tucson, Arizona, United States of America; 3 Center for Insect Science, University of Arizona, Tucson, Arizona, United States of America; Arizona State University, UNITED STATES

## Abstract

Honey bees (*Apis mellifera*) collect and store both honey and pollen in preserved forms. Pollen storage involves the addition of honey or nectar and oral secretions to pollen granules. It is controversial whether the duration of pollen storage alters the palatability or nutritive value of the pollen storage medium. We examined how bees utilize different-aged stored pollen during an extended pollen flow. The deposition of pollen into wax cells and subsequent consumption were monitored daily on 18 brood frames from 6 colonies over an 8d observation period. Despite a greater abundance of older stored pollen cells on brood frames, bees showed a marked preference for the consumption of freshly-stored pollen. Two to four day-old pollen cell contents were significantly more likely to be consumed, while pollen cell contents more than seven days old were eaten at much lower rates. Similar experiments that controlled for cell abundance and spatial effects using cage assays yielded the same result. One day-old stored pollen was consumed approximately three times more often than 10d-old stored pollen, and two times more often than 5d-old stored pollen. These consumption preferences for freshly-stored pollen occurred despite a lack of clear developmental advantages. Young adult workers reared for 7 days on 1d-, 5d-, or 10d-old stored pollen showed no difference in body mass, stored pollen consumption, hindgut fecal material accumulation, or hypopharyngeal gland (HPG) protein titers, suggesting that different-aged pollen stores did not vary in their nutritional value to adult bees. These findings are inconsistent with the hypothesis promoting a period of microbially-mediated, “beebread maturation” that results in greater palatability or nutritive value for aged pollen stores. Rather, stored pollen that is not eaten in the first few days accumulates as excess stores preserved in a less preferred, but nutritionally-similar state.

## Introduction

As a highly eusocial insect, the honey bee *Apis mellifera* maintains large populations across periods of food scarcity by bulk preservation and storage of food materials [1,2]. Used to fuel metabolism, stored honey is overwhelmingly antimicrobial and combines many different properties favorable for food preservation. Pollen, which is the primary source of protein, lipids, sterols, minerals, and most vitamins for bees, is stored in nest cells that are accessible to all adult workers in the colony [1,3–5]. During its formation, stored pollen (also known as bee bread) is altered by the addition of nectar, honey and glandular secretions [6–8]. Anther pollen from flowers is collected and coated with regurgitated nectar to form corbicular pollen during transit. Pollen foragers carry an excess of honey in their honey stomach (crop) to “glue” pollen grains together into pellets during foraging trips [9]. Corbicular pollen pellets are tightly packed into cells and further coated with additional layers of nectar, honey, and oral secretions. By some estimates, stored pollen in cells eventually contains 40–50% simple sugars [8]. Both corbicular pollen and stored pollen differ from anther pollen in that individual pollen grains are immersed in a sugar rich acidic matrix consisting of regurgitated honey and oral secretions. [8–11].

All collected pollen is packed into storage cells before consumption by colony members. Young adult workers consume over 80% of all stored pollen to complete their adult development and redistribute pollen to other bees in the form of nutrient rich secretions and semi-processed pollen fragments [12–16]. Young adults require large amounts of pollen to develop secretory glands (hypopharyngeal glands, or HPG) and nutrient reserves that are critical for later roles as nurses and foragers [5,15,17,18]. Although pollen is not absolutely required for individual bee survival, bees deprived of pollen sources have significantly shortened lifespans and reduced task efficiences. Compared to pollen-fed adults, pollen-starved adult workers differ in their fundamental gene expression and show poor development of hypopharyngeal glands (HPG) used to produce food secretions to feed others [19]. Individual workers that are absolutely deprived of pollen may exhibit stunted growth [20], reduced internal nutrient stores [21], reduced nursing ability [22,23], and a generally shortened lifespan [24]. Under severe pollen deprivation, workers cannot efficiently nurture the next generation of young larvae due to an inability to produce larval jelly. Colonies lacking immediate pollen resources can consume excess eggs or larvae (brood cannibalism), or fall back on the internal nutrient stores of adult workers as a reserve [5,17,25–27]. However, such emergency resources rarely sustain a colony more than a couple of worker generations.

Largely unknown is how honey bees consume stored pollen as pollen stores accumulate and age in the colony. Numerous authors have speculated that stored pollen undergoes significant microbially-driven nutrient changes during the initial phases of storage [3,6,28–31]. According to this “beebread maturation” hypothesis, stored pollen is fermented by colony microbes into a more palatable, nutritionally-superior form either through biosynthesis of new nutrients (i.e. vitamins) or greater bioavailability of refractory nutrients (i.e. amino acids) [3,30]. Bees would be predicted to consume older stored pollen after a period of maturation to obtain maximal nutritional benefits. However, recent studies by Anderson and coworkers found that bees preferentially consume freshly-deposited stored pollen over older stored pollen [10]. This result, combined with the observation that microbial activity decreases significantly in stored pollen following five days of storage, questions the assumed roles of microbes in stored pollen [10].

In this study, we examined how bees utilize different-aged stored pollens in the colony environment. We first tested honey bee consumption preferences for different-aged stored pollen produced over 8 days of storage. We focused on feeding preferences of young adult workers because these bees are overwhelmingly the primary consumers and redistributors of pollen resources for the colony [3,12–16,32,33]. Adult workers have been shown to vary consumption rates in response to nutritive, phagostimulant, and toxicological factors in the pollen [8]. Since different-aged pollens varied considerably in their abundance and spatial distribution on comb, we also examined consumption preferences in cage assays that controlled for these factors.

A key prediction of the beebread maturation hypothesis is that older stored pollen is nutritionally superior to freshly-stored pollen. Because dietary pollen quality impacts adult development and function, we examined worker growth on pollen stored for different durations. We also examined the development of a tissue whose growth is known to depend on pollen availability. HPG produce proteinaceous glandular secretions used to feed developing larvae and other adult bees [12–14]. HPG are formed during early adult development (0d to 10d) and are largely reabsorbed by the body during the nurse-to-forager transition [15,34]. We used HPG to gauge the suitability of different-aged pollen sources in producing functional nurse bees.

## Materials and methods

### Honey bee colonies and floral forage

We used honey bee colonies headed by Italian queens (*Apis mellifera linguistica*) for all experiments and collections. All colonies were established in April 2014 from 1.4 kg worker bee packages headed by Italian queens (C.F. Koehnen and Sons, Glenn, CA). Each colony was maintained in a 10 frame single Langstroth deep box with a top feeder, entrance reducer, and bottom board (Brushy Mountain Bee Farm, Moravian Falls, NC, USA). Colonies were kept in an apiary at the eastern edge of the Red Rock Agricultural Center (RRAC; University of Arizona College of Agriculture and Life Sciences Agricultural Experimental Station, Pinal County, Arizona, USA; 32.553669, -111.320481, 569m elevation) from colony establishment (April 2014) through use in field experiments (mid- to late-August 2014). The RRAC apiary was located in a Lower Sonoran desert creosote bush community consisting of rangeland bisected by drainage ditches and cattle pools [35]. This moderately-grazed site was dominated by creosote (*Larrea divaricata*), triangle bursage (*Ambrosia deltoidea*), mesquite (*Prosopis velutina*), and acacia (*Acacia* spp.), with the latter two predominantly located along dry washes and pools.

Colonies were carefully managed to limit exposure to agrochemicals from distant irrigated fields. Agricultural fields used primarily for cotton farming were located at the main research station (minimally 4.7 km) and distantly along the Santa Cruz River (minimally 6.8 km). However, no agricultural crops or weeds were in bloom at the main research station from colony establishment through the experimental period. Frequent observations of colony foragers revealed that workers foraged almost exclusively on native plants within 240m of the apiary during the month leading up to and including the experimental period. Workers engaged in water foraging gathered untreated surface water and/or ground water from ephemeral puddles, a water tank in the apiary, and a cattle pond located 380m from the apiary. Pesticide analysis of food stores from these colonies after the experiment and from colonies at this site in subsequent years revealed an absence of agrochemicals aside from Varroa mite treatment residues from previously-used comb. No chemical treatments were applied to the colonies from the time of package establishment through the end of the experiment. Representative samples of stored pollen and stored nectar/honey were removed from the colonies and analyzed for pesticide contents by the National Science Laboratory (USDA-Agricultural Marketing Services, Gastonia, North Carolina, USA). 3 g of each material were pooled from 120–150 cells in 6 colonies. No pesticides aside from trace levels of the miticides Amitraz and Thymol were detected in these food materials in full pesticide panel screenings.

Colonies were amply provided with food store frames and supplemental sugar and pollen before the experiment to support worker nutrition and limit foraging on distant agricultural fields. All colonies had excess stores of pollen and nectar present for at least three weeks prior to and through the experiment. Colonies were further supplemented with pollen patty (1:1:1 mixture (w/w) of dried corbicular pollen (Great Lakes Pollen Mix, Great Lakes Bee Supply, Galesburg, Michigan, USA), MegaBee pollen supplement and sucrose (MegaBee, San Diego, California, USA)) and 2:1 sucrose:water sugar syrup from the start of the mid-summer dearth in early July 2014 up until 1 week before the experiment. By the beginning of the experimental period in mid-August 2014, each colony contained approximately 22,000 to 26,000 adult workers, 5–7 frames of brood, and at least 2200 cells of stored pollen. Colonies slightly increased brood production and net pollen stores through the 8d experimental period.

Experiments were conducted in mid-August 2014 during the summer monsoon after four weeks of significant rains. Colony pollen collection was characterized by direct observation of foragers and monitoring of four sentinel colonies that were similar in size, food stores, and bee populations to the experimental colonies. Colony foragers collected polylectic mixtures of pollens that included at least 13 distinct plant species as determined by direct observation of foragers between the colonies and flowers (Table 1). Colonies experienced a moderate pollen flow from creosote and mesquite three weeks before and through the experimental period. Numerous summer ephemeral plants were also visited by the bees through the experimental period. The total amount of corbicular pollen collected daily varied considerably between sentinel colonies but was remarkably consistent on a day-to-day basis for individual colonies over the 8d experimental period. Average daily collections of corbicular pollen from individual sentinel colonies ranged from 12 g/day to 26 g/day as detected by front loader pollen traps (Brushy Mountain Bee Farm, Moravian Falls, NC, USA). However, for each individual sentinel colony, the amount of corbicular pollen collected on each day was within 18% of the overall colony average over the 8d period. Pollen foraging occurred primarily (96%) during the morning from just before sunrise (0515 hours) to mid morning (1000 hours). By late morning (after 1000 hours), most foragers had switched to water foraging to cool the colony from the intense desert heat. All sampling and colony manipulations were conducted from late morning to early afternoon (1000 to 1300 hours) to avoid disruption of pollen foraging activities.

**Table 1:** Plants visited by honey bee foragers originating from the sentinel colonies at the RRAC experimental apiary (Pinal County, AZ, USA).

Species	Family	Common name
*Acacia constricta*	Fabaceae	Whitethorn acacia
*Acacia greggii*	Fabaceae	Catclaw acacia
*Amaranthus spp*.	Amaranthaceae	Amaranth spp.
*Atriplex polycarpa*	Amaranthaceae	Cattle saltbush
*Boerhavia intermedia*	Nyctaginaceae	Spiderling
*Ferocactus wislizeni*	Cactaceae	Fishhook barrel cactus
*Geraea canescens*	Asteraceae	Hairy desert sunflower
*Hymenothrix wislizeni*	Asteraceae	Trans-Pecos thimblehead
*Larrea tridentata*	Zygophyllaceae	Creosote
*Lepidium thurberi*	Brassicaceae	Thurber's pepperweed
*Parkinsonia florida*	Fabaceae	Blue palo verde
*Proboscidea wrightii*	Martyniaceae	Devil's claw
*Prosopis juliflora var*. *velutina*	Fabaceae	Velvet mesquite

Foragers were observed flying directly between the four sentinel colonies and these flowering plants. All colonies had foragers visit and manipulate the flowers of these plants. All plants were within 240m of the colonies. [35, 36]

### Preparation of single-aged stored pollen comb sections

Experiments 2 and 3 (described below) required pollen comb sections containing stored pollen of uniform age. These sections were obtained by restricting colony pollen deposition to a single frame for a limited time period (24h). Stored pollen was acquired from 6 colonies containing 20,000 to 32,000 workers and 4–7 frames of brood. Bees were housed in hive equipment consisting of (top to bottom) a top board, a top feeder, a Langstroth deep box (a “brood box” that contained all brood and initial food stores; internal dimensions 23.65 cm H x 47.31 cm L x 37.47 cm W), 10 fully drawn Langstroth deep frames (48.26 cm L x 23.18 cm H), an entrance reducer, and a bottom board (Brushy Mountain Bee Farm, Moravian Falls, North Carolina, USA).

Pollen deposition was concentrated on comb sections by directing the access of pollen foragers to a single frame. First, an empty Langstroth deep box (“pollen deposition box”) was placed between the brood box and the bottom board. To keep returning foragers from entering the brood box, a rectangular wire mesh barrier with worker-excluding openings (3.2 mm x 3.2 mm openings, 48.3 cm L x 38.1 cm W) was placed between the upper brood box and the lower pollen deposition box. A single Langstroth deep frame containing empty cell wax comb (“pollen deposition frame”) backed by Plasticell foundation (Brushy Mountain Bee Farm, Moravian Falls, NC, USA) was placed in the pollen deposition box to provide empty cells for pollen deposition by the foragers. A second frame completely filled with nectar and capped honey stores was placed next to the pollen deposition frame to provide a sugar source for the foragers. Bee escapes were placed on the corners of the brood box to allow workers to leave to forage but prevent their return to the brood box (Brushy Mountain Bee Farm, Moravian Falls, North Carolina, USA). Returning foragers entered the colony through the entrance formed by the pollen deposition box, entrance reducer, and bottom board and deposited their corbicular pollen in cells on the pollen deposition frame. Pollen deposition occurred under these restricted conditions during a 24h period from 1000 hours to 1000 hours the following day. However, the bulk of the pollen deposition (over 96%) occurred in the early morning (0515 to 1000 hours) near the end of the 24h collection period.

Once the pollen deposition period ended, the single-aged stored pollen frame was placed in a hive configuration that greatly limited further pollen deposition and oviposition. The pollen deposition box containing the single-aged stored pollen frame was moved to the top of the brood box. Further pollen deposition and oviposition on the single-aged stored pollen frame was restricted by the placement of a rectangular wire mesh barrier with queen excluding openings (4.1 mm x 4.1 mm openings, 48.3 cm L x 38.1 cm W) between the upper pollen deposition box and the lower brood box. This barrier selectively allowed workers to tend the stored pollen but prevented the passage of queens or additional corbicular pollen into the pollen deposition box. Single-aged stored pollen frames were stored in this manner until use in bioassays.

Single-aged stored pollen frames were cut into uniform-sized comb sections to provide food choices of standardized size and deposition age to bees in bioassays. Each frame was carefully cut with heavy shears into rectangular 8 cm H x 5 cm W sections before use in bioassays. Each comb section contained about 90 to 120 stored pollen cells out of the approximately 160 cells present. Honey and nectar cells on the comb were emptied by pipette and then cleaned of residues by selective exposure of cell contents to sugar-deprived workers.

### Experiment 1: Formation and emptying of different-aged stored pollen cells on colony frames

The deposition and emptying (consumption of cell contents) of stored pollen in storage cells was monitored by marking pollen cell changes on a transparency sheet placed over the frame face on a daily basis [10]. During the first monitoring time point (beginning of day 1 observation period), three frames with pollen stores and brood were selected from each of 6 colonies. Adult bees were removed from each frame and all stored pollen cells were traced with a black circle by Sharpie pen. Each frame was replaced in the colony and re-examined on subsequent days between 1000 and 1300 hours to track changes in stored pollen deposition and removal. Later changes in stored pollen cell contents were marked daily on each frame face’s transparencies over 8 days using a different color Sharpie pen on each day. The formation of a new pollen cell was indicated with a circle, while the complete removal of a cell’s contents was indicated with an X. The total number of cell contents deposited and emptied on each frame during each 24h period was therefore enumerated on a daily basis. We restricted our observations to an 8d period to limit the number of repeated colony disturbances. However, all monitored colonies continued to rear eggs and larvae and deposit pollen despite these frequent disturbances.

We readily recognize that this method of cell monitoring is highly conservative in that it only detects changes in pollen content at the whole cell level and underestimates small changes in individual stored pollen cells [10]. Full stored pollen cells contain up to 150 mg of pollen, while individual bees deposit no more than several mg of corbicular pollen nor consume more than a few mg during a feeding event (Winston, 1989). Pollen deposition and consumption events by individual bees were therefore largely undetectable at this observation scale. The “age” of pollen cells thus represents a maximum time since the first significant pollen deposition occurred (referred throughout the manuscript as the pollen’s age), and is in fact a mosaic of different-aged pollen layers created by numerous workers. We also acknowledge that this method may overcount deposition and removal of recently filled cell contents since newer cells have had less time to be completely filled by individual corbicular pollen deposits. To limit this effect, we only counted cells as “full” if stored pollen covered the entire bottom of the cell and “empty” if the cell bottom was completely emptied.

During the eighth and final monitoring time point (end of day 7 observation period), the total number of new stored pollen cells deposited (cells filled since the previous day’s observation) and emptied were tabulated by cell age for all monitored frames. Stored pollen cells marked on the first day with a black circle (8d^+^) were at least 8d-old, but were otherwise indeterminately older. All other stored pollen cell contents were deposited during a known time interval and were referred to by their maximum possible “age” of pollen deposition from 1d-old to 7d-old.

After the last marking time point, we calculated the number of stored pollen cells created and emptied (cell contents removed) during the last 24h interval. This set of markings was the only time point for which all stored pollen age classes were fully represented. The absolute numbers of stored pollen cells present, newly-formed (freshly deposited), and emptied (cell contents consumed) were tabulated for each of the eight age groups (1d-old to 8d^+^-old pollen cells) during this final interval.

Although absolute cell counts detail which age-classes of stored pollen are predominantly eaten, changes in absolute cell counts do not accurately indicate actual preferences for a given pollen age. More abundant pollen types (by cell count) would likely be consumed in greater numbers than less abundant pollen types based simply on their higher abundance on the frames. To obtain an accurate understanding of consumption preferences, we needed to correct for the relative abundance of each stored pollen age class. We calculated the relative pollen cell emptying rate (RPCER) for a given pollen age-class as:

$$\frac{\left(number\;of\;pollen\;age\;class\;cells\;emptied\right)\left(total\;number\;of\;pollen\;cells\;present\right)*100\%}{\left(number\;of\;pollen\;age\;class\;cells\;present\right)\left(total\;number\;of\;pollen\;cells\;emptied\right)}$$

or ((the observed fraction of age-class pollen cells emptied)/(the expected fraction of age-class pollen cells emptied)) x 100%.

This metric readily detects over-emptying or under-emptying (values above or below 100%) of stored pollen cell age classes relative to the values expected if pollen cell emptying was random with regard to stored pollen age. No value could be calculated for emptying of 0d to 1d-old stored pollen since these stored pollen cells were emptied before their deposition was detected.

### Experiment 2: Adult worker preferences for different-aged stored pollens

One problem with our field assessments of worker stored pollen age preferences is that different-aged stored pollens vary in their abundance and spatial distribution on colony frames. Bees foraging for stored pollen on colony frames encountered many more older stored pollen cells than newer stored pollen cells based on numbers alone. To address these concerns, we conducted a series of dual choice assays where caged bees were presented with two different pollen comb sections of approximately uniform stored pollen cell age, size, and abundance.

Dual choice assays were conducted with 3d- to 4d-old (post-emergence) adult workers because adult pollen consumption increases sharply just before this age [15]. Each choice assay replicate was conducted in a Plexiglas and wire mesh cage measuring 15 cm H x 9 cm W x 6 cm D. Workers were transferred into cages as newly-emerged bees to limit handling stress. Newly-emerged adult workers were obtained from six colonies headed by unrelated Italian queens (C.F. Koehnen and Sons, Glenn, CA). Young adult workers were collected within 12h of emergence from capped brood frames maintained in an environmental room at 32°C and 35% relative humidity. Workers from different colonies were combined in equal proportions to each cage to provide similar levels of worker genetic variation among cages. Approximately 90 workers were placed in each Plexiglas cage and provisioned with 20 mL 1:1 sugar syrup and 20 mL deionized water bottles. These bottles were placed upside down through cap-sized holes in the ceiling of the cage. A small strip of wax foundation (extending from the floor to the ceiling) was embedded into the back of the cage wall to provide easy access to the sugar and water sources. Bees were reared through the third day on mixed-aged sections of stored pollen comb. These pollen sources were provided in excess to avoid overcrowding during feeding. All cages were maintained in an environmental chamber in the dark at 30°C and 35% relative humidity.

In the dual choice assay, bees were given a choice between two different single-aged pollen comb sections (prepared as previously described). Each 8 cm H x 5 cm W comb section was placed vertically against the side wall such that the two sections were separated by the width of the cage. Bees were allowed to feed *ad libitum* on the two pollen sources for 20 hours. Each comb section was weighed before and after the assay. The total pollen consumed from each source was calculated as the difference between the pre- and post-assay masses of the source. Six cage replicates were performed for each of the three choice comparisons (1d-old vs. 5d-old pollen cells, 1d-old vs 10d-old pollen cells, and 5d- vs. 10d-old pollen cells).

### Experiment 3: Development of young adult workers fed different-aged stored pollens

The development of young workers reared exclusively for 7 days on a single-aged stored pollen source was compared across three single-aged stored pollen treatments (1d-, 5d-, or 10d-old stored pollen). Newly-emerged adult workers were collected from caged capped brood frames over an 8h period. Approximately 60 newly-emerged workers were placed in a Plexiglas cage as previously described. Each cage was provisioned with 20 mL 1:1 sugar syrup and 20 mL deionized water bottles. Four cage replicates were established for each of the three pollen treatments. All cages were maintained in an environmental chamber in the dark at 30°C at 35% relative humidity. Sugar syrup bottles were changed daily to avoid fermentation.

The bees in each cage were provided with a pre-weighed pollen comb section containing either 1d-, 5d-, or 10d-old stored pollen (previously described). Each pollen source was provided in excess (90 to 120 pollen cells) to avoid overcrowding during feeding. The pollen source of each cage was replaced daily with new, preweighed material. The amount of pollen consumed was calculated as the difference between the pre-weight and post-weight masses of each pollen source.

At the end of 7 days, the bees were anaesthetized with carbon dioxide and three bees were randomly selected from each cage for analysis of adult development. To obtain a more accurate measurement of worker body mass, we removed the highly variable gut contents of the honey stomach (crop), hindgut, and rectum before weighing the remainder of the body. The contents of these gut sections consist largely of food materials (sugar solutions (honey stomach) or feces (hindgut/rectum)) that otherwise inflate whole body measurements of worker mass. The whole body mass (wet weight) was measured immediately after removal of these contents. Hindgut and rectal fecal contents then were measured separately to provide an index of the amount of pollen consumed by the bee during adult development. Hindgut and rectal fecal contents have previously been used to estimate relative consumption of solid foods since adult workers do not defecate inside their nest (i.e. the cage) but accumulate feces in their hindgut and rectum [15]. The fecal contents were dried at 60°C for 48 hours and weighed to determine the mass of unexcreted, indigestible fecal material present after pollen digestion.

Each bee head was then dissected and chemically analyzed to assess HPG development. Gland development was quantified as the total soluble protein content of macerated HPG tissue, a metric commonly used as a proxy for HPG size and quality [16,37]. Soluble protein content was quantified by analysis of homogenized extracts with a BCA assay (Pierce Biotechnology, St. Louis, MO, USA). Freshly-dissected HPG were homogenized in 1000 μL PBS buffer by 30 mg of 1.0 mm diameter zirconium beads in a Bead Beater (BioSpec Products, Inc., Bartlesville, OK, USA) for 30 seconds. The resulting homogenate was centrifuged for 5 minutes and 25 μL of the supernatant was analyzed by the kit protocol. Soluble protein content was calculated by comparison against a standard curve generated by an albumin standard.

### Statistical analysis

Consumption biases towards different-aged pollen stores were detected by comparing the observed consumption rate with the rate expected under random consumption. The overall distribution of emptied and unemptied cells across pollen-age classes was compared against the numbers expected by random emptying (consumption of cell contents) alone by a chi square test of independence[38]. Post-hoc pairwise comparisons of emptied and unemptied cell distributions between pollen-age class were examined with a chi-square test of independence. Pairwise comparisons were made with 2 x 2 contigency tables with emptied/unemptied cells as one variable and pollen-age class as the other variable. Chi-square critical values were comparied against a Bonferroni-corrected significance level (p = 0.05/number of pairwise comparisons (21), or 0.00238) t.o correct for the large number of pairwise comparisons In the dual choice assays, the amounts of each pollen source consumed by the caged bees were compared by a paired t-test. In the no choice performance assays, adult worker body mass, pollen consumption (fresh mass consumed), dried hindgut contents, and HPG protein titers were compared across pollen treatments by a one-way ANOVA. Means from significant comparisons were compared by a post-hoc Tukey’s test. All consumption and bee development metrics were tested for normality with the Shapiro-Wilk test, Kolmogorov-Smirnov test, and by examination of normality probability plots. All statistical comparisons were performed with SAS 9.2 (SAS Institute, Cary, NC). All data sets used in the statistical analyses and results figures were included in a supplemental file (S1 File).

## Results

### Experiment 1: Formation and emptying of different-aged stored pollen cells on colony frames

**Fig 1:**
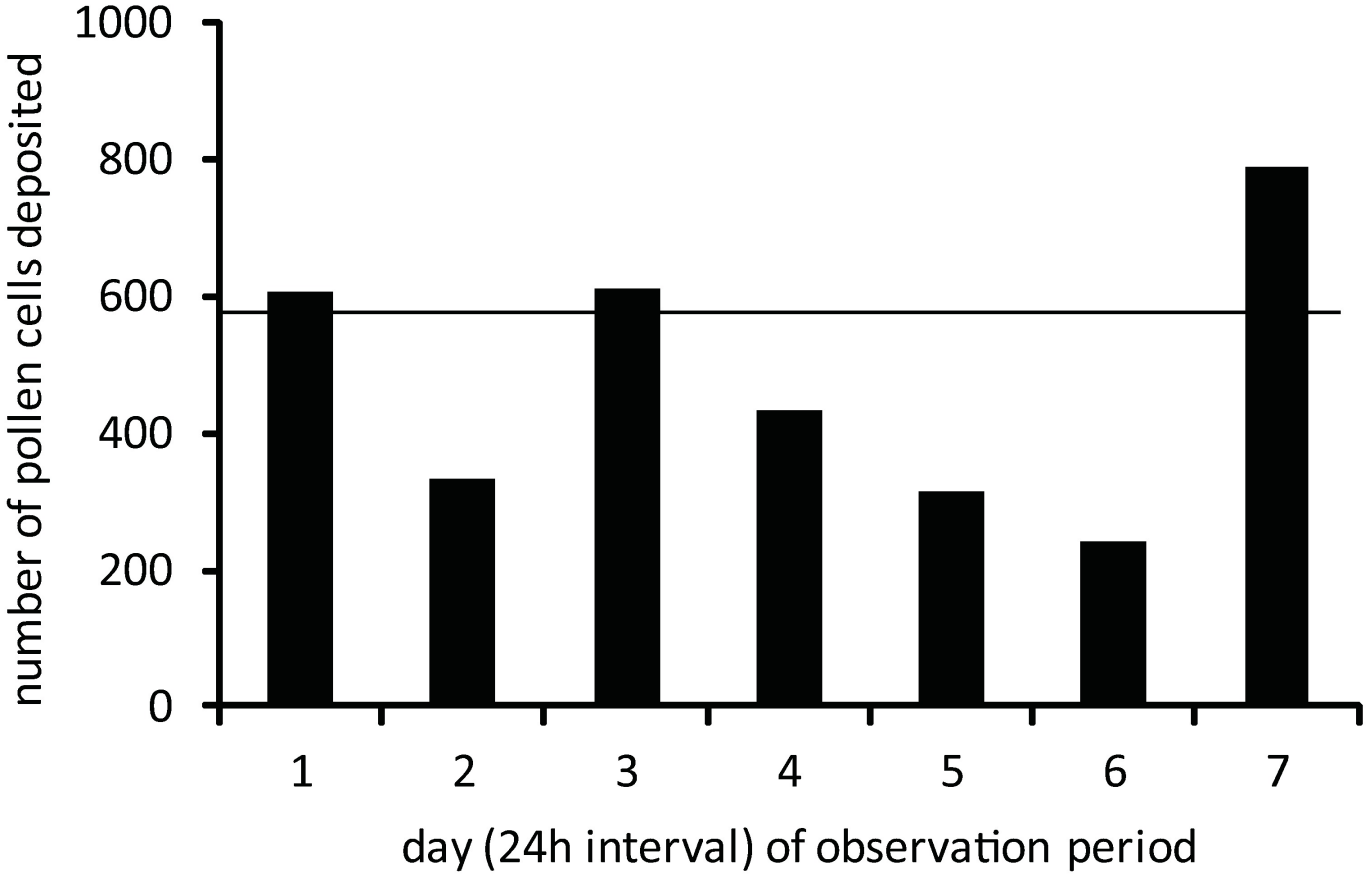
Total number of new stored pollen cells formed daily in six colonies during the 8d frame monitoring period. The line indicates the average number of new stored pollen cells deposited daily in all colonies over the 8d observation period.

**Fig 2:**
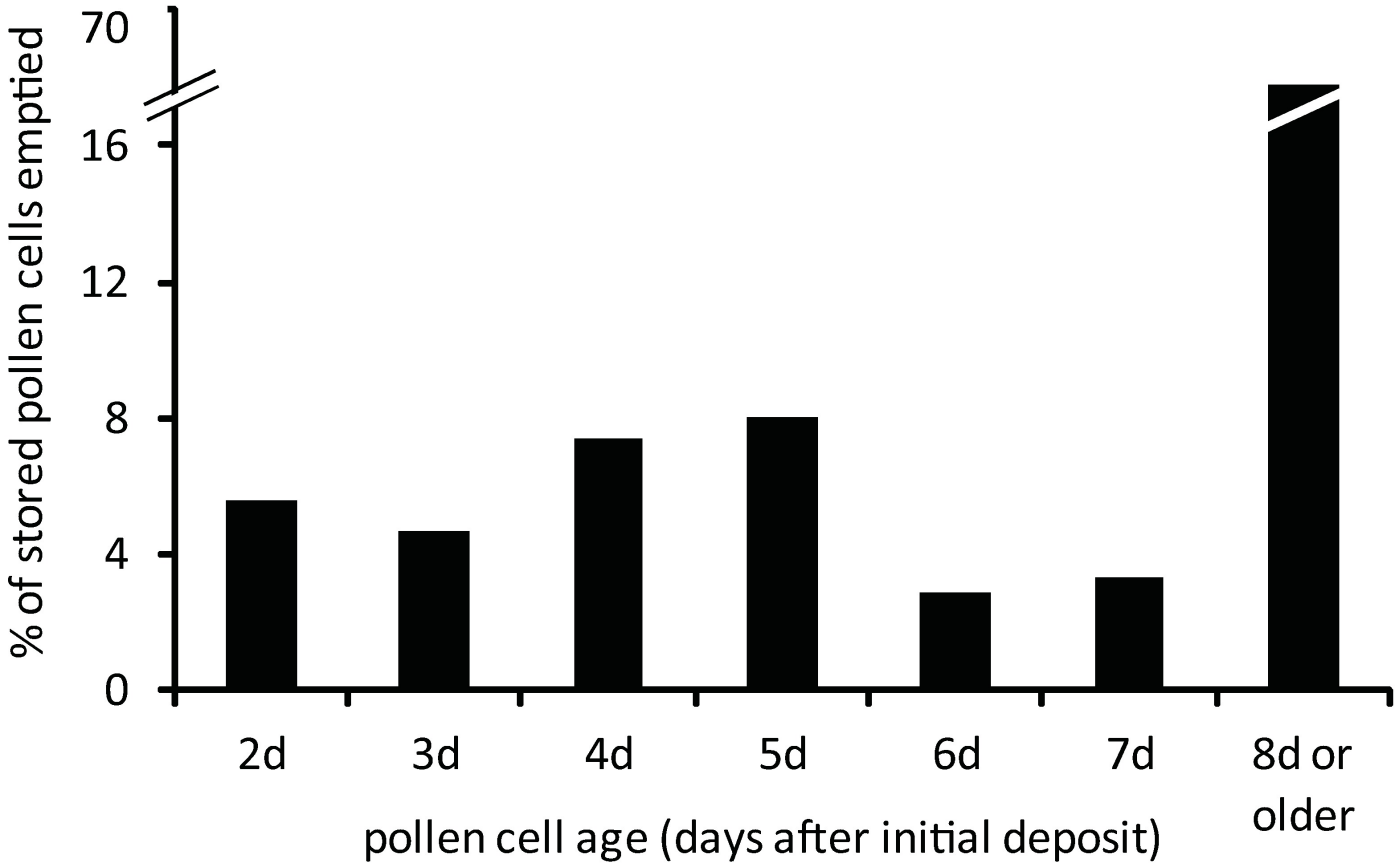
Proportions of different-aged pollen cells emptied during the last observation interval. Stored pollen cell “age” refers to the earliest time of pollen deposition in the cell and therefore represents a maximum time since pollen deposition. The emptying of stored pollen cells formed in the last 24h interval (1d-old stored pollen cells) was undetectable and not included in the analysis. In total, 774 stored pollen cells were emptied in the last interval.

Honey bee colonies experienced moderate turnover in stored pollen cells relative to the pollen stores originally available on the selected frames (S1 File). 10,637 stored pollen cells were initially present in the 18 frames monitored from the six colonies. Collectively, 3,326 (31.2% of initial cell count) new stored pollen cells were formed and 2,159 (20.3%) stored pollen cells were emptied (contents completely consumed) for a net gain of 1,171 (11.1%) stored pollen cells over the 8 day monitoring period. Bees deposited on average 554.3 ± 58.1 SE (5.2%) new stored pollen cells/colony during the observation period, but new stored cell formation varied considerably from day to day (Fig 1, S1 File). Overall, bees completely emptied an average of 359.8 ± 78.9 SE (3.4%) percolony during the observation period. At the level of individual colonies, the net influx of pollen varied considerably between colonies, with four colonies experiencing net gains and two colonies experiencing net losses of stored pollen cells over the period. Remarkably, we detected only 4 cases where stored pollen cells were completely emptied and refilled during the entire observation period. In terms of absolute pollen consumption, colony bees largely subsisted on the contents of superabundant (80.7% of stored pollen cells counted in the experiment) 8d^+^ older stored pollen cells. In the last observation interval, older stored pollen cells comprised 68.2% of the stored pollen cells emptied of their contents (Fig 2, S1 File). All other cells formed during the 8 day period accounted for 31.8% of the stored pollen cells completely emptied during the last observation interval. Clearly, the large scale consumption of the contents of older pollen cells was primarily due to their high abundance on the frame.

**Fig 3:**
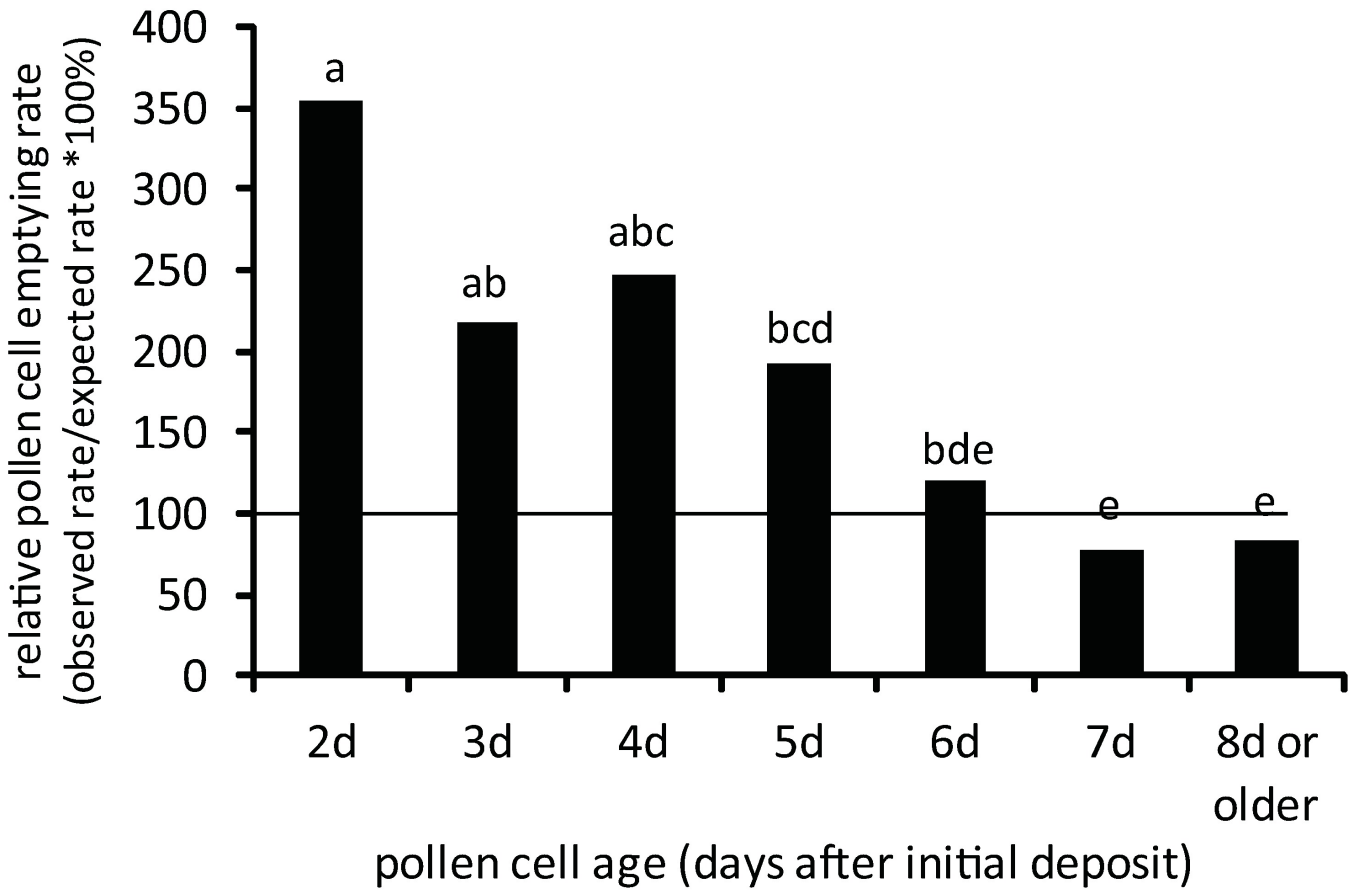
Relative pollen cell emptying rate (RPCER) of different-aged stored pollen cells from colony frames during the last observation interval. For each pollen-age class, the observed numbers of emptied and unemptied cells was compared against the numbers expected by pollen-age class abundance and overall emptying rate alone. Pollen-age classes sharing the same superscript did not significantly differ in their relative proportions of emptied and unemptied pollen cells as determined by post-hoc pairwise comparisons (chi-square test of independence, Χ^2^_total_>9.323, df = 1, p < 0.00238 (Bonferroni-corrected significance level)).

However, a different pattern of stored pollen consumption emerges among bees when the relative abundance of different-aged stored pollen cells is factored in. Bees emptied different-aged stored pollen cells at different rates (Fig 3, S1 File; chi square test of independence, Χ^2^_total_ = 215.364, df = 6, p<0.00001). Bees displayed significantly higher emptying rates of freshly-deposited stored pollen cells when cell emptying rates were adjusted for cell abundance on the frames. 2d-, 3d-, and 4d—old stored pollen cells were fully emptied at levels significantly higher than 7d-old and 8d-old stored pollen cells (Fig 3, S1 File; chi-square test of independence, Χ^2^_total_>9.323, p<0.00238 (Bonferroni-corrected significance level)). These newer stored pollen cells were fully emptied at rates 193% to 354% above predicted levels. By contrast, 7d-old and 8d^+^-old stored pollen cells were emptied at lower rates relative to their abundance. A critical change in cell content consumption patterns occurred between the 5d- and 6d-old stored pollen cells. Intermediate-aged 5d-old and 6d-old pollen cells were neither over-emptied nor under-emptied but appeared to represent a transitory phase in attractiveness to bees foraging for food within the colony.

### Experiment 2: Adult worker preferences for different-aged stored pollens

**Fig 4:**
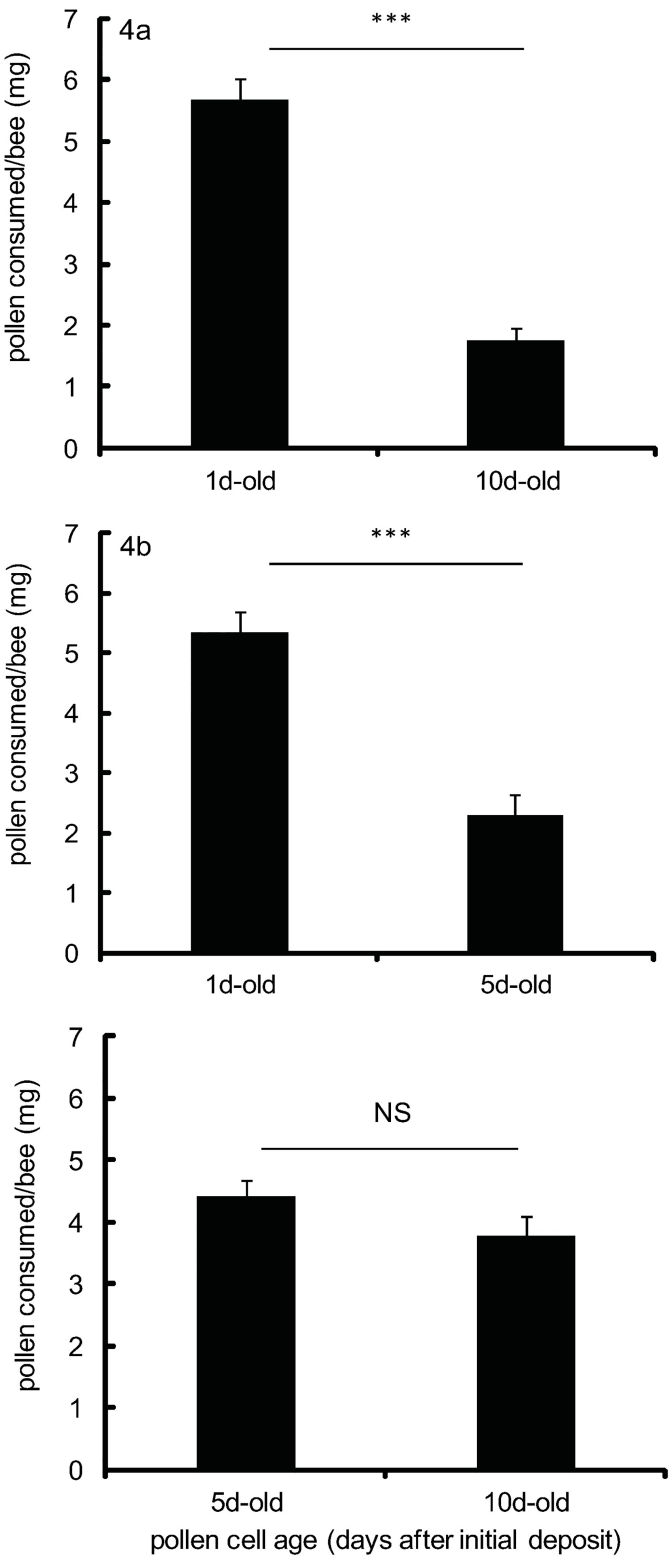
Young adult worker consumption of different-aged pollens ((a) 1d-old vs. 10d-old, b) 1d-old vs. 5d-old, c) 5d-old vs. 10d-old) presented in a dual choice assay. * The amount of each stored pollen type consumed by cage workers varied significantly (*** p<0.0001) or did not vary significantly (NS) by a paired t-test (p<0.05). Error bars are S.E.

In dual choice assays, 3d- to 4d-old workers were allowed to freely feed on two different-aged comb sections containing either 1d-, 5d-, or 10d-old stored pollen cells. Workers preferentially consumed new 1d-old stored pollen over both older age-classes of stored pollen. Bees ate 3.4x as much 1d-old stored pollen as 10d-old stored pollen (Fig 4a, S1 File; paired t-test,t = 11.96, df = 5, p<0.0001) and 2.3x as much 1d-old stored pollen as 5d-old stored pollen (Fig 4b, S1 File; paired t-test, t = 5.31, df = 5,p<0.0001). However, 5d-old stored pollen was not consumed significantly more than 10d-old stored pollen (Fig 4c, S1 File; paired t-test, t = 0.96, df = 5,p = 0.3797).

### Experiment 3: Development of young adult workers fed different-aged stored pollens

**Fig 5:**
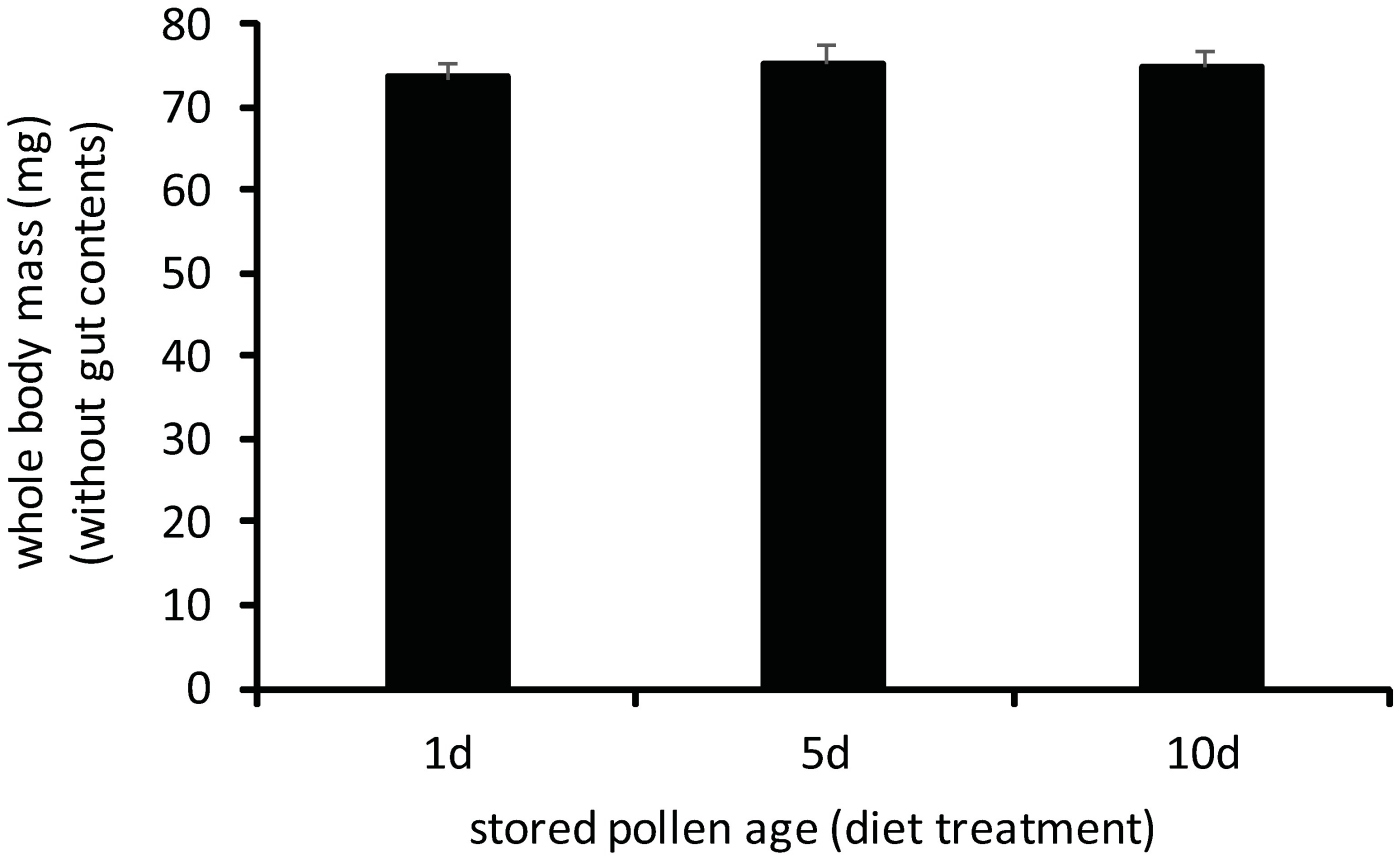
Whole body mass (fresh mass without gut contents) of adult workers reared exclusively for 7d on 1d-old, 5d-old, or 10d-old stored pollen. Treatment means did not vary significantly by a one-way ANOVA. (p<0.05; error bars are S.E.).

**Fig 6:**
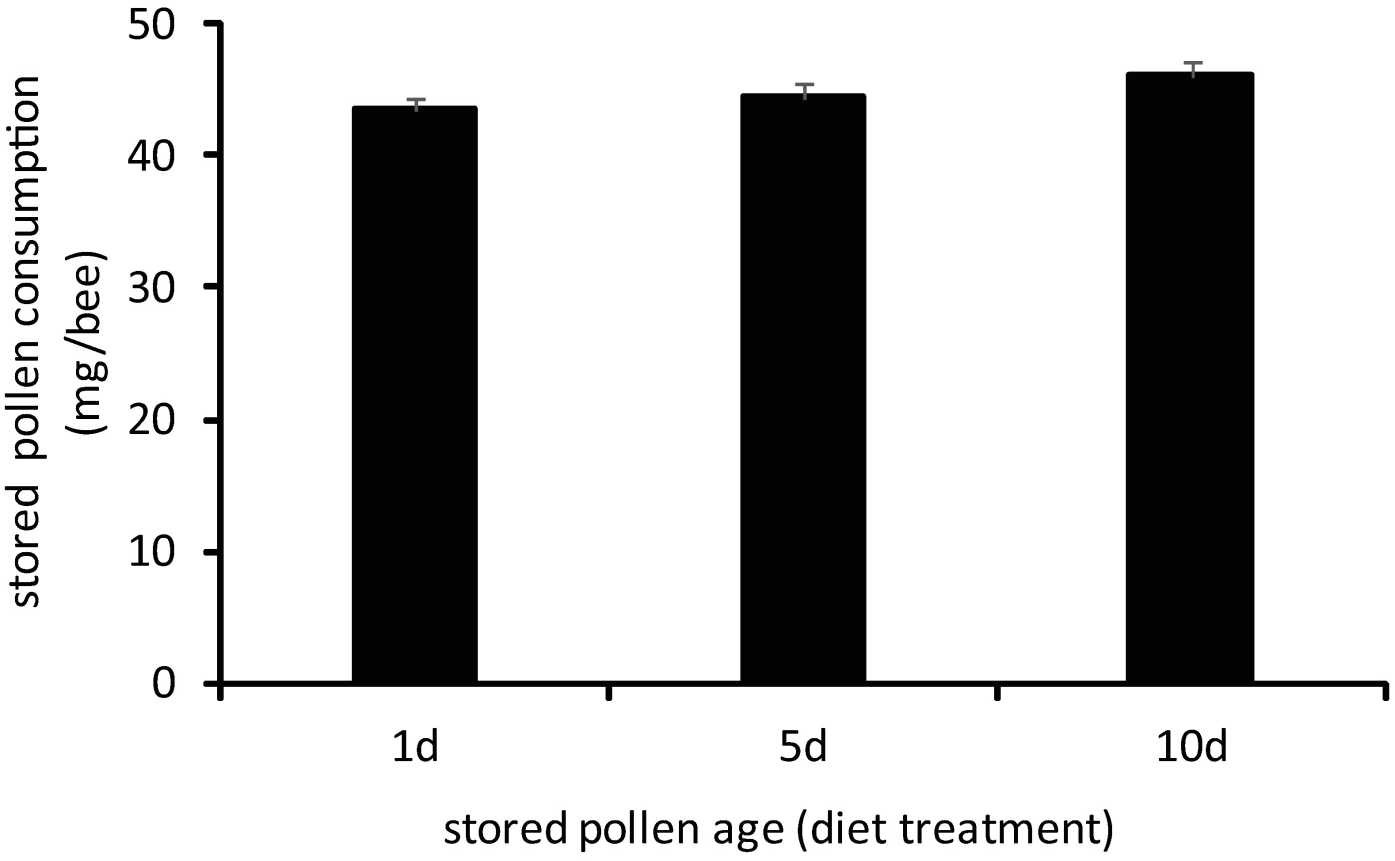
Average stored pollen consumption (fresh mass) of adult workers reared exclusively for 7d on 1d-old, 5d-old, or 10d-old stored pollen. Treatment means did not vary significantly by a one-way ANOVA. (p<0.05; error bars are S.E.).

**Fig 7:**
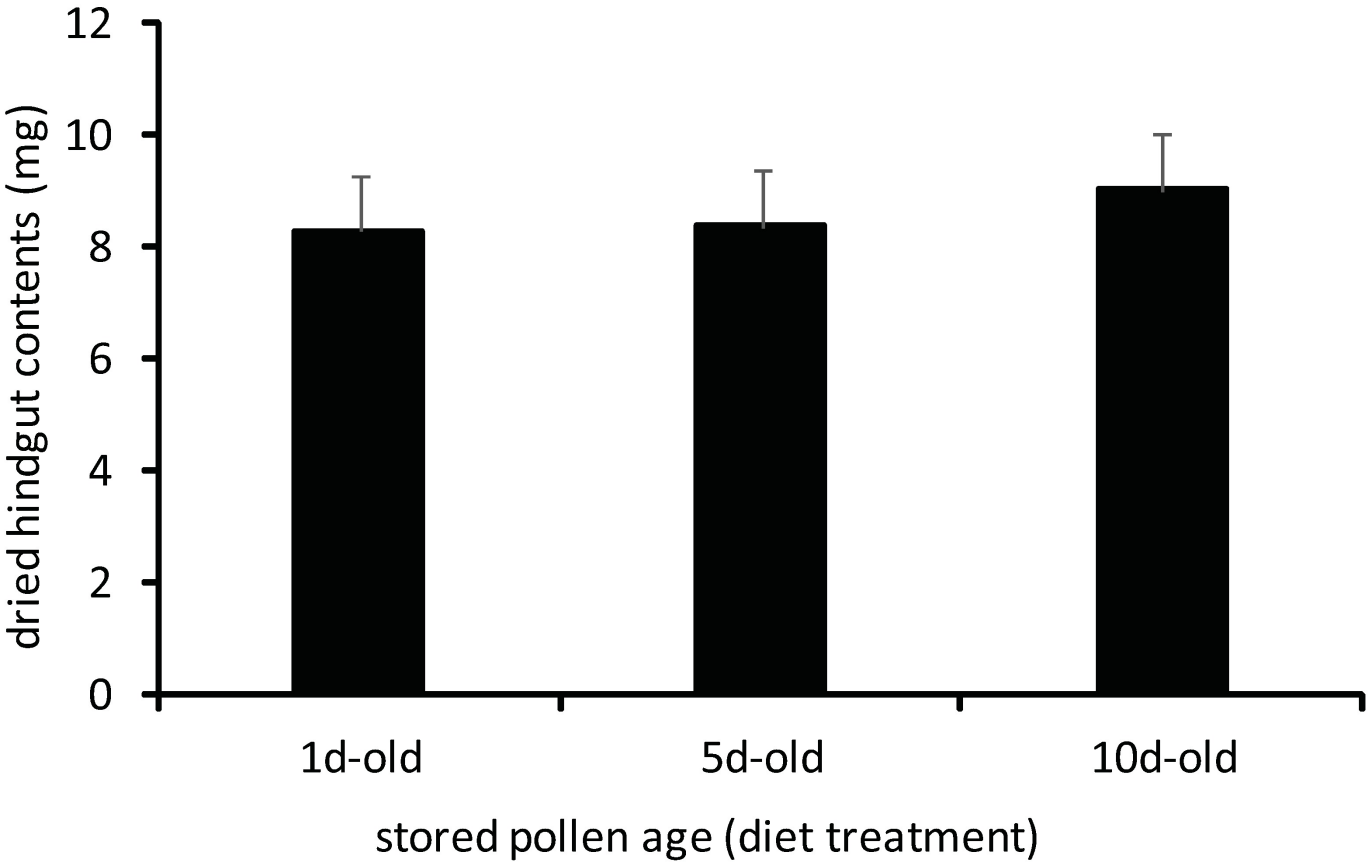
Dried hindgut fecal masses of adult workers reared exclusively for 7d on 1d-old, 5d-old, or 10d-old stored pollen. Treatment means did not vary significantly by a one-way ANOVA. (p<0.05; error bars are S.E.).

No differences in developmental performance and consumption were observed in young adult workers reared for 7 days exclusively on 1d-, 5d-, or 10d-old stored pollens. Worker whole body mass did not significantly vary in bees fed different-aged stored pollens (Fig 5, S1 File; one-way ANOVA, F = 0.34, df = 2, p = 0.7129). No differences were observed in worker consumption of different-aged stored pollens as measured by two metrics. Workers consumed similar fresh weight amounts of stored pollen, regardless of stored pollen age (Fig 6, S1 File; one-way ANOVA, F = 0.56, df = 2, p = 0.5701). Sampled workers fed different stored pollens also had similar amounts of pollen-derived material in their hindguts (Fig 7, S1 File; one-way ANOVA, F = 0.37, df = 2, p = 0.6935). Hindgut fecal mass, which consists primarily of indigestible or poorly digested remnants of pollen grains, has been previously used as a metric of stored pollen consumption by individual adult workers ([15, but see [39]). The absence of differences in both metrics of stored pollen consumption suggests that all three stored pollen sources induced similar consumption patterns when choice was restricted.

**Fig 8:**
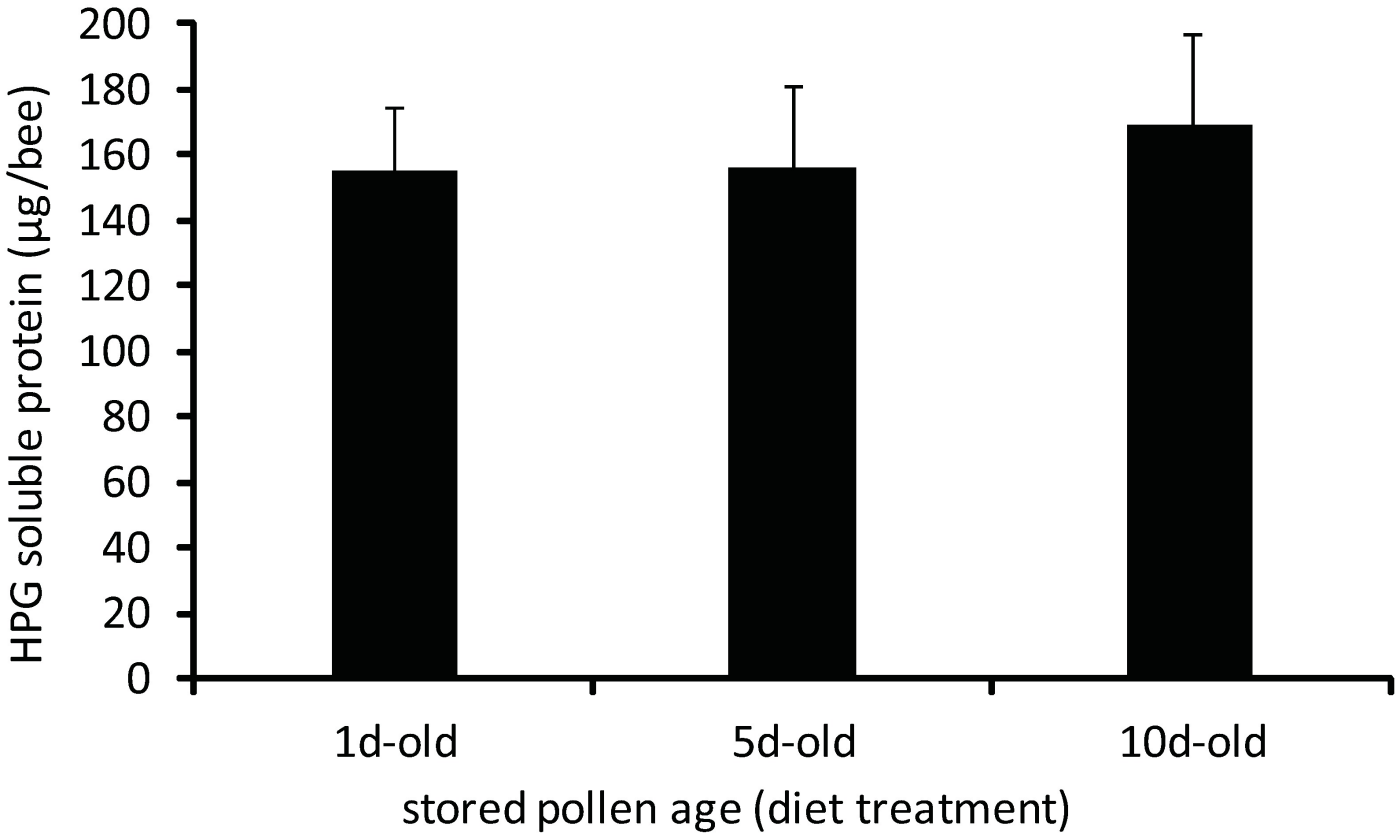
Total soluble protein contents of HPG from adult workers reared exclusively for 7d on 1d-old, 5d-old, or 10d-old stored pollen. Treatment means did not vary significantly by a one-way ANOVA. (p<0.05; error bars are S.E.).

Young workers fed different-aged stored pollens also experienced similar development of HPG over the 7d developmental period, as shown by soluble protein titers. Soluble protein titers of the HPG were not significantly different for bees fed different-aged stored pollens (Fig 8, S1 File; one-way ANOVA, F = 0.18, df = 2, p = 0.8483). These results demonstrate that these workers received sufficient nutrition for development of HPG protein reserves, irrespective of diet.

## Discussion

Honey bees rapidly preserve pollen during the initial phases of storage by the addition of hygroscopic honey, nectar, and oral secretions [6–8,10,39]. The freshly-formed composite of pollen, nectar, and oral secretions is briefly more attractive than older stored pollen, yet retains its nutritive value to bees over moderate storage periods. This pattern is precisely the opposite pattern of consumption that would be expected if stored pollen needed to mature before consumption by workers. Our results clearly show that no latent period of beebread maturation is required to render stored pollen palatable or nutritious to bees. On the contrary, the consumption patterns observed here suggest that freshly-stored pollen is consumed within days of deposition. The older pollen stores that accumulate represent excess pollen cells that were not consumed before becoming less attractive. Rather than maturing to a palatable form, older stored pollen sources are only likely to be fully consumed once freshly-stored pollen sources are largely exhausted. Likewise, our performance trials do not support the concept of alteration by beneficial microbes resulting in a nutritionally superior product. Recently, Anderson and coworkers indicated that stored pollen lacks an active core bacterial community as predicted by the beebread maturation hypothesis [10]. In two recent studies, Anderson and coworkers [10] and Saviara and coworkers [40] noted considerable environmental variation in stored pollen bacterial community composition. Furthermore, both studies found little overlap between bacterial communities in stored pollen and honey bee guts. Critically, the absence of a viable stored pollen bacterial community militates against active functions for bacteria during beebread storage.

Many researchers appear to have underestimated the timing and rate of key physiochemical changes associated with pollen preservation [28, but see 10]. Part of the problem may be a misunderstanding of the time frame required for critical microbial action to occur. While honey bees clearly prefer fermented over non-fermented food materials, the microbial activity responsible for beebread fermentation does not appear to primarily occur during pollen storage. Recently, Anderson and coworkers found that microbial activity in stored pollen was very low initially, and decreased sharply within only five days of storage [10]. Most of the key preservative properties (i.e. acidification, hygroscopic media, prooxidant enzymes) appear to be already active in the nectar and oral secretion components prior to their application in pollen storage [8,10,41]. Thus, critical microbial contributions to pollen preservation occur during the conversion of dilute nectar to stored forms (i.e., well before collection of the first pollen grain).

Despite marked bee preferences for freshly-stored pollen, stored pollen age does not appear to affect bee growth and development. Young bees restricted to less preferred ages of pollens consume equivalent amounts of stored pollen and develop similarly to bees fed preferred stored pollens. Curiously, the strong feeding preferences for freshly-stored pollen over older stored pollen did not result in reduced consumption in bees reared solely on the less preferred diet. Bees restricted to older stored pollen diets consumed similar amounts as bees fed newer stored pollen diets. The absence of performance differences hints at the great flexibility of stored pollen as a preserved food. Our results suggest that medium term storage of pollen does not carry a developmental penalty to future worker generations. Bees may preferentially consume freshly-stored pollen, yet upon its disappearance, may also consume less-preferred older pollen stores and experience similar levels of adult growth. Our observations agree with studies that have found little change in the dietary value of well-maintained stored pollen [37].

While our results indicate that stored pollen retains its dietary value to bees for several days, it does not resolve questions of the value of storage on pollen nutrient quality. The relationship of pollen processing to pollen nutrient content is particularly contentious, especially regarding bioavailability of essential nutrients [7,8,11]. Most important, fundamental differences between the abilities of bees and chemists to extract nutrients from refractory materials make nutrient comparisons difficult. Pollen itself is somewhat nutritionally refractive, although bees can digest nutrients through fractures and holes in indigestable materials [8,11]. However, the sharp reduction of microbial activity shortly after storage militates against extensive post-storage nutrient changes [10]. A more critical, but underappreciated, factor in stored pollen nutrition may be dilution of pollen with less nutritious materials (Nicolson’s “mixture problem”), such as nectar, honey, and water [7,8,11].

Our results clearly indicate that pollen storage age also had little effect on young adult hypopharyngeal gland development and retention. The full development of HPG on different-aged stored pollens indicates that the bees receive adequate amounts of protein from stored pollens over a range of storage times [18,36,42]. These absences of differences in growth and consumption may be due in part due to the relatively higher nutritional quality of polyletic pollen mixtures. In a comparison of local Arizona pollens, Schmidt and coworkers found that a polylectic pollen mixture similar to that used in our assays strongly promoted high consumption rates and bee survival [43] compared to most monolectic pollen sources. Honey bees generally perform better on polyfloral diets than monofloral diets [44–46].

At this point, we should iterate that an absence of growth and development costs associated with moderately-older pollen stores does not necessarily apply to long-term pollen storage. Most of the pollen stores in our colonies had accumulated since the end of the mid-summer dearth (locally from mid-June to late-July) [35]. Our local colonies consumed almost all of their pollen stores that they had collected during the early summer during the mid-summer dearth. As a result, stored pollen would have been 4 to 5 weeks old at the oldest. In contrast, long-term pollen stores have been strongly associated with poor consumption, reduced growth performance, and increased mortality, especially if the cells are not well maintained. Maes and coworkers (2016) recently found that workers reared on older (21d) stored pollen experienced reduced adult growth, increased mortality, and development of a dysbiotic gut microbiome compared to workers reared on more recently (14d) stored pollen [47]. Previous authors have noted that bees may not perform well on poorly-maintained stored pollen that has been stored for months [10,37]. The effects of consuming degraded pollen may bee particularly deleterious during extended periods of high colony stress, such as overwintering [48]. Under marginal conditions, colony food preservation may erode as hygienic behavior declines, environmental control of the colony periphery is reduced, and water and microbes infiltrate stored pollen. Under such an absence of preservation, the chemical composition of stored pollen may change and render the pollen unsuitable for consumption. Eventually, bees may respond to suboptimal stored pollen by entombing the degraded food cell with propolis [49].

How these feeding preferences contribute to mechanisms that regulate colony pollen use has yet to be explored. As individuals in a colony, honey bees must balance activities that result in pollen collection, pollen consumption, and brood rearing [50]. Honey bees encounter highly variable amounts of pollens of different botanical origin and nutritional value both inside and outside of the colony [8, 11,27,51]. Honey bee workers select for these pollens twice—once as foragers outside the colony and once as nest bees during consumption of stored pollen. Workers collectively alter pollen consumption, nutrient redistribution (brood rearing and trophollaxis), and pollen collection (foraging) activities in response to pollen availability and brood population size [27, 50, 52–56]. Foragers have shown a generalized, though not absolute, ability to distinguish between floral species based on visual, olfactory, phagostimulant, and nutrient cues [37, 57–62]. Recent studies have suggested that honey bee and bumblebee foragers can select for floral resources that complement nutritional imbalances within the colony [59, 62, 63]. Honey bee foragers therefore can respond to changing pollen stores and consumer demand by modulating forager effort, time as a forager (the nurse-to-forager transition), and selection of high quality pollens [50, 52, 64–69].

While forager activities partially correct imbalances in pollen supply, worker feeding preferences shape the use of pollen once it is stored within the colony. However, the role of pollen consumer preferences in colony pollen utilization is less understood than foragers. Young nest workers that consume most stored pollen have different responses and olfactory sensitivities towards food materials than older workers, an attribute thought to contribute to age-related polyethism [70–75]. The pollen feeding experiences of young adult workers may also inform foraging preferences later in life [70, 76]. However characterized, worker feeding preferences for different-aged stored pollens may impact colony access to stored pollen, a resource whose availability and quality changes through the year. Individual colonies vary considerably in their turnover of stored pollen, with much higher rates of consumption during fixed periods of population growth than the rest of the year [27]. These pollen consumption preferences may also have strong genetic components. For example, Africanized bees generally convert a much greater proportion of pollen stores to brood than “hoarder” subspecies, such as European bees [56, 77, 78].

It remains to be demonstrated if the cues underlying these observed preferences are generalized cues or specific to this foraging system. Nonetheless, we have observed feeding preferences for fresh pollen in two locations on different types of forage at different times of the year [10]. A key feature of social insect signaling systems is the simplification and convergence of a complex set of possible cue candidates to common signals [79–81]. Social insect food preservation strongly parallels this trend regarding the convergence of critical properties, which may include the microbial communities, chemical environment, and storage conditions of honey bee colonies [10]. Honey bee processing of food materials may be an example of this trend, as food materials from thousands of botanical sources are reduced to a common hygroscopic, antimicrobial matrix. The common factor that honey bees use to achieve these conditions of food storage is honey, a key antimicrobial present in all honey bee colonies [8,10,48]. Behaviors that alter consumption preferences and consumption rates of pollen in the colony directly govern how colonies use the food that is available [50]. Given that discerning young adult consumers complete pollen utilization initiated by older foragers, honey bee feeding preferences toward stored pollen impact nothing less than the food balance of the colony as a whole.

## Supporting information

S1 File Data sets used in statistical analyses and results figures of stored pollen cell counts, worker pollen preferences, and worker performance on different-aged stored pollens. (XLSX)
