# Correction: Honey bees preferentially consume freshly-stored pollen

**DOI:** 10.1371/journal.pone.0249458

**Published:** 2021-03-25

**Authors:** Mark J. Carroll, Nicholas Brown, Craig Goodall, Alex M. Downs, Timothy H. Sheenan, Kirk E. Anderson

The fourth author’s name appears incorrectly. The correct name is: Alex M. Downs.
